# Psychometric Measures of Instruments of Occupational Performance in Children With Developmental Coordination Disorder: A Systematic Review

**DOI:** 10.1111/cch.70327

**Published:** 2026-08-06

**Authors:** Marcela Favilla, Carolina Barbosa de Souza, Jorge Alberto de Oliveira, Flávio Rebustini, Renata Hydee Hasue

**Affiliations:** ^1^ Faculty of Medicine University of São Paulo São Paulo Brazil; ^2^ School of Physical Education and Sport University of São Paulo São Paulo Brazil; ^3^ School of Arts, Sciences and Humanities University of São Paulo São Paulo Brazil

**Keywords:** daily activities, developmental coordination disorder, reliability and validity, test validity

## Abstract

**Background:**

Because instruments are routinely employed by health professionals, it is essential to establish their psychometric quality, particularly regarding validity evidence.

**Objective:**

The study aimed to analyse the psychometric measures used in studies of validity evidence of instruments for assessing the occupational performance of children with developmental coordination disorder.

**Methods:**

A systematic review was conducted according to PRISMA (Preferred Reporting Items for Systematic Review and Meta‐Analysis) guidelines using five electronic databases.

**Results:**

Twenty‐four articles were included, with 17 different scales. Discriminant validation was the most reported psychometric measure, with half of the studies reporting using parametric and nonparametric analyses. Among the psychometric methods verified in the studies, reliability analysis was the most prevalent, using Cronbach's alpha and test–retest; one study used ordinal alpha. One third of the studies used exploratory or confirmatory factor analysis for internal structure.

**Conclusion:**

The validation techniques used varied among the studies found, and these were sometimes incomplete and confusing, as well as an insignificant result in relation to the use of more contemporary concepts for the types of validity evidence. Factor analysis for internal structure was rarely reported in the studies; however, it stands out for being a more robust technique for this type of validation.

## Summary

- Assessment of occupational performance/daily activities is a central component in the therapeutic process.
- Quality of assessment instruments depends on good psychometric methods.
- More instruments with updated methods of evidence of validity are needed

## 1 Introduction

Neurodevelopmental disorders, which include autism spectrum disorder (ASD), attention‐deficit hyperactivity disorder (ADHD) and developmental coordination disorder (DCD), are very common health conditions during childhood, and this topic has been widely discussed in the literature. DCD can affect about 5% to 6% of school‐aged children (Gomes and Sirigu 2015; Karkling et al. 2017), and it is also common for this disorder to be present in ASD and ADHD.

Children with motor development disorders, specifically DCD, have impairments in the acquisition and development of age‐appropriate motor coordination skills, even with adequate opportunities, impacting the child's ability to perform daily tasks and participate in social and academic activities (APA 2013; Zwicker et al. 2012).

According to DSM‐5 criterion B for the diagnostic of DCD, ‘the deficit in motor skills in Criterion A significantly and persistently interferes with age‐appropriate daily activities (e.g., self‐care and self‐maintenance), impacting academic/school productivity, pre‐professional and professional activities, leisure, and play’ (APA 2013). This condition can negatively impact performance in activities of daily living (ADLs), such as dressing and undressing, eating and school‐related activities (Gomes and Sirigu 2015), affecting engagement in their occupations (Karkling et al. 2017). Therefore, DCD is considered a neurodevelopmental disorder that impacts children's occupational participation and performance.

According to the American Occupational Therapy Association (AOTA 2020), ADLs, instrumental activities of daily living (IADLs), rest and sleep, education, work, play, leisure and social participation are the main human occupations. Considering that children's main occupations include the ability to perform daily activities and participation in social and school contexts, child assessment should be comprehensive and consider aspects such as task performance and participation in leisure activities. It is worth highlighting that understanding occupational performance is central to the practice of occupational therapy.

Assessment is a central component in the therapeutic process, contributing to the formulation of interventions and in measuring their results. According to AOTA (2020), assessment is not just an initial step but a process that underpins the planning and implementation of interventions. Through it, it is possible to know the individual's ability to participate in their activities and also identify strengths and those that need improvement. Assessments that integrate these dimensions tend to provide a more holistic view of the child's development and the necessary interventions. Likewise, the assessment process can allow us to understand the motor and functional implications on performance in their occupations. In the case of DCD, motor difficulties in tasks are characterised as one of its diagnostic criteria.

Despite the need for instruments adapted to children, it is assumed that there are still few instruments that have been developed specifically to assess performance in tasks that are part of the family, school and social context, especially with a focus on motor skills of children with DCD or other pathologies. Furthermore, it is questionable to what extent the psychometric methods used to develop existing instruments that assess these demands provide good evidence of validity.

Current recommended psychometric methods, according to The Standards for Education and Psychological Testing (AERA 2014), include five steps for health assessment instruments development: (1) test content; (2) response process; (3) internal structure; (4) relationship with other variables; (5) consequence of the test. These steps for evidence of validity have replaced versions that were based on the pretest, construct validity and criterion validity, as they were termed by Consensus‐based Standards for the selection of health Measurement Instruments (COSMIN) (Mokkink et al. 2010).

Test content examines whether the items of an instrument adequately and comprehensively represent the construct intended to be measured; response process examines how participants understand, interpret and respond to the items; internal structure evaluates whether the organisation of the items reflects the theoretical dimensions of the construct; relationship with other variables investigates the association of the instrument with external measures or expected outcomes; and consequences of the test considers the effects and practical impacts of its application (AERA 2014).

Each type of validity evidence has a set of techniques that can be used and have been refined over time. The challenge lies in knowing when to use each of them. There is no longer room for the use of outdated techniques, as they do not align with good scientific practices, becoming an ethical issue (Ferretti‐Rebustini 2023; Borsboom 2006, 2022).

Because the use of instruments is part of the practice of health professionals, it is necessary to guarantee their quality with regard to their evidence of validity and, consequently, the scientific evidence base for their practices. According to Pasquali (2009), psychometrics is defined as the science that aims to interpret individuals' responses to a series of tasks (items). It has a trajectory of advances in recent decades, supported by two strands: classical test theory (CTT) and item response theory (IRT) (Sartes and Souza‐Formigoni 2013). Although CTT focuses on the reliability of the total test score, IRT provides a more detailed analysis of individual item properties, such as difficulty and discrimination, allowing for more precise measurement.

Such evidence analyses the relevance and representativeness of the parts of an instrument so that it can achieve its purpose. Therefore, the selection of an assessment tool for occupational performance of children depends on both the scope and the psychometric properties of the instrument. For this reason, the objective of this study was to analyse the psychometric measures used in studies on the validity evidence of instruments for assessing the occupational performance of children with DCD.

## 2 Methods

### 2.1 Research Strategy and Source of Study Selection

This systematic review followed the PRISMA guidelines. Two independent researchers used six electronic databases covering the areas of health and psychometrics (PubMed, CINAHL, Bireme [BVS], Embase, Scopus and Web of Science). The selected databases were chosen for their multidisciplinary scope. Articles that addressed original research on psychometric studies were selected. A time frame of 20 years was used, including publications between 2004 and 2024, published in any location. All articles were published in English or Portuguese (Brazil) language. The search was conducted in August 2024.

### 2.2 Study Selection and Data Extraction Process

Searches were conducted using the following Boolean descriptors and tags: (Transcultural Studies OR Cross Cultural Comparison OR Validation Study OR Validation Studies OR Psychometric OR Psychometrics) AND (Child OR Children OR Children with Disability OR Children with Disabilities OR Child developmental) AND (Activities of Daily Living OR ADL) AND (Motor Skills OR Motor Skills Disorder OR Motor Skills Disorders OR Developmental Coordination Disorder OR Developmental Coordination Disorders). The search terms were selected as they best approximated and encompassed articles that aligned with the proposed objective. This study has no conflicts of interest.

The Rayyan software was used to facilitate the organisation and selection of articles, as well as collaboration between the two reviewers. The review followed the following steps: (1) formulation of the research question; (2) search for articles by descriptors in the databases and extraction of titles; (3) exclusion of duplicate titles; (4) reading of titles and selection of abstracts; (5) reading of abstracts and selection of articles for full reading; (6) reading of articles in full; (7) extraction of data for descriptive synthesis. Discrepancies were discussed and resolved between the two reviewers until consensus.

Articles adapted into other languages were retained; however, they were published in English. After the initial reading and selection, the articles were read in full for synthesis, including the scale format and the psychometric measures for each identified instrument; at this stage, articles were also excluded when they did not appropriately meet all inclusion criteria.

The instruments were analysed and subsequently discussed following the recommendations for sources of evidence established in The Standards for Educational and Psychological Testing (AERA 2014) and in Survey Scales—A Guide to Development, Analysis and Reporting by Johnson and Morgan (2016).

### 2.3 Study Eligibility Criteria

The following criteria were considered for inclusion of articles: (1) validation studies of instruments that assessed occupational performance; (2) population assessed were children, focusing on school‐age children; (3) assessment of children's performance covering different disabilities or focusing on DCD; (4) instruments that contemplated conditions related to participation in activities and tasks, such as ADLs, school tasks, functional skills, motor skills, play and social roles.

The following articles were excluded: (1) review studies; (2) validation articles of instruments for specific conditions, such as cerebral palsy, rheumatoid or neuromuscular disease, as well as classification measures; (3) articles conducted exclusively with a sample of preschool children (0–3 years of age) and over 12 years of age, without including children under that age.

### 2.4 Data Analysis

Data were collected according to the MOOSE (Meta‐Analysis and Systematic Review of Observational Studies in Epidemiology) guidelines (Stroup et al. 2000). From the studies selected and read in full, the following data were extracted for descriptive analysis: general and content characteristics of the selected instruments and psychometric measures reported in the studies (types of validity evidence and methods of analysis used).

## 3 Results

### 3.1 Study Selection

**FIGURE 1:**
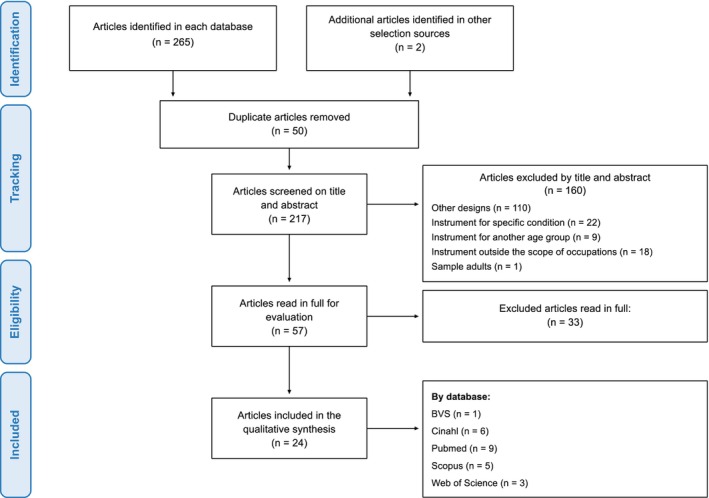
Flowchart of the study selection process.

The electronic literature search returned 265 studies in the databases. After removing 50 (18.8%) due to duplication, 215 (81.1%) articles were reviewed to verify the eligibility criteria in the titles and abstracts. After reviewing the titles and abstracts, 160 (60.3%) articles were excluded. The full‐text review phase excluded another 33 (12.4%) articles, leaving 22 articles from the electronic search included in this review. The manual search produced two more articles. Thus, 24 articles were included and synthesised. The PRISMA (Preferred Reporting Items for Systematic Review and Meta‐Analysis) diagram shows this process in full (Figure 1).

### 3.2 General and Content Characteristics of the Selected Instruments

The findings identified 17 different scales reported in the 24 articles. Furthermore, articles providing evidence of validity for instruments assessing children's occupational performance were published between 2007 and 2023 and originated from 13 countries. High‐income countries, including Spain, the United States, Australia, Taiwan, Sweden, Germany, Austria, Switzerland, Canada, the Netherlands and France, comprised 70.8% (*n* = 17) of the scientific production that validated instruments for assessing children's performance.

The DCD (DCDDaily) (Montes‐Montes et al. 2020; Delgado‐Lobete et al. 2020; van Der Linde et al. 2013; van der Linde et al. 2014) and Paediatric Evaluation of Disability Inventory (PEDI) (Schulze et al. 2014, 2016; Dumas et al. 2012; Mancini et al. 2016) instruments were reported in 16.7% (*n* = 4 each) of the total studies, followed by the Developmental Coordination Disorder Questionnaire (DCDQ) (Ferreira et al. 2019; Prado et al. 2009; Moraes et al. 2022) in 12.5% (*n* = 3).

Performance in ADLs was the latent variable most reported by the studies (58.3%; *n* = 14), followed by coordination (focusing on performance in activities and tasks) with 41.7% (*n* = 10) and social participation (25.0%; *n* = 6). It is worth noting that in this search for instruments, the latent variable ‘occupational performance’ included a set of variables, which could include instruments that used other terms, referring to children's performance in activities and tasks that are part of their daily lives, or participation, functional skills and competencies related to this.

Different methods of measuring children's performance were identified, as well as different types of scale formats. Scale formats between 3 and 9 points were identified (example, 1. Regularly, 2. Sometimes, 3. Seldom, 4. Not yet/never), and assessment included the level of difficulty of the task, quality of performance, frequency of performance, level of assistance received and level of responsibility. The questionnaire format and the target audience of parents and caregivers as respondents were predominant (83.3%; *n* = 20). The type of application varied between caregiver self‐administered and interview. The articles also reported four instruments that were based on direct testing or observation of the child and one with a combined method. The number of questions per instrument ranged from 10 to 442.

### 3.3 Psychometric Measures Described in the Selected Studies

Table 1 presents the types and methods of validity evidence reported in the validation process of the instruments according to each study. The terms used in the tables were the same as used in the original articles. Among the types of validity evidence reported, discriminant (54.1%; *n* = 13) and construct (50%; *n* = 12) were the most prevalent. It was observed that one study (0.24%) analysed face validity (Ramalho et al. 2013). Among the 24 studies found, four (16.6%) were versions cross‐culturally adapted to the Brazilian population: DCDQ‐BR, LC‐MABC‐2, LDCDQ‐BR and PEDI‐CAT‐BR (Mancini et al. 2016; Ferreira et al. 2019; Moraes et al. 2022; Ramalho et al. 2013).

**TABLE 1:** Types of validation and methods reported in the analysis of the instruments evaluated in the selected articles.

Author, year	Instrument (acronym)	Face validity	Content validity	Discriminant validity	Construct validity	Predictive criteria	Competitor criteria	Psychometric validity measures reported
Noreau et al. (2007)	LIFE‐H	☐	☑	☐	☐	☐	☑	Short version—Content validity: panel of 29 judges, professionals and parents of children with functional limitations; qualitatively and quantitatively analysed. Criterion validity, correlated with PEDI and WeeFIM, using Pearson's coefficient. Intra‐ and inter‐rater reliability, using ICC.
Brantner et al. (2009)	MAND	☐	☐	☑	☐	☐	☑	Concurrent validity with the MABC instrument: Spearman correlation; discriminative accuracy was calculated using the sensitivity, specificity and positive and negative predictive values tests.
van Duijn et al. (2009)	VABS	☐	☐	☐	☑	☐	☐	Cronbach's alpha used for reliability analysis; inter‐rater reliability and test–retest, ICC used. Principal component analysis (PCA) for factor analysis, KMO and Bartlett's test.
Bagley (2010)	ASKp38	☐	☐	☐	☑	☐	☐	Mplus software, to confirm the dimensionality of the conceptual model; exploratory factor analysis (EFA), Tucker–Lewis index. Dimensional model, confirmatory factor analysis (CFA) and item response, using Rasch model with Winsteps, differential item functioning (DIF).
Chien et al. (2012)	ACHS	☐	☐	☐	☑	☐	☑	Construct validity: Rasch method, FACETS software used, item response validity. Concurrent validity: correlation with DCDQ and VABS, Spearman's correlation coefficient used.
Chien and Brown (2012)	CHSQ	☐	☐	☐	☑	☐	☑	Internal construct validity: Rasch model (unidimensionality test; DIF; person response validity; test segmentation). For external construct: correlation analysis with the ACHS and VABS instruments.
Dumas et al. (2012)	PEDI‐CAT	☐	☐	☑	☐	☐	☐	Discriminant validity for comparing groups: *t*‐test used; *F*‐test and Satterthwaite's were used to calculate variance. ICC for test–retest reliability analysis.
Munkholm et al. (2012)	School AMPS	☐	☐	☐	☑	☐	☐	FACETS software, Many‐Facet Rasch Model (MFR) analysis, SEM, Cronbach's alpha. The reliability coefficient was corrected using the Spearman‐Brown formula.
Bult et al. (2013)	APCP	☐	☐	☐	☑	☐	☐	Cronbach's alpha was used to analyse internal consistency; test–retest reliability was calculated using ICC.
Ramalho et al. (2013)	LC‐MABC‐2	☑	☑	☑	☑	☐	☑	An expert panel composed of four translators, three of whom were PhDs, used CVI and the Wilcoxon and Friedman tests for content validity (clarity and relevance). Face validity was carried out on the target audience (20 parents, 20 classroom teachers, 10 physical education teachers and 10 physiotherapists). Construct validity: convergent correlations in the EMT and discriminant validity the Wilcoxon test and the Kruskal Wallis test, Mann–Whitney *U* post hoc tests. Cronbach's alpha for internal consistency.
van Der Linde et al. (2013)	DCDDaily	☐	☑	☑	☐	☐	☐	Content validity: literature review carried out by six professionals; discussion on the relevance of activities of daily living (ADLs) by nine professionals in the field, resulting in a pilot version; pilot study on 35 children. Test–retest and inter‐rater reliability, calculated by ICC; internal consistency measured by Cronbach's alpha. Concurrent validity: calculated by Spearman's correlation; discriminant validity: comparison between groups calculated by the Mann–Whitney test.
Lin et al. (2014)	CFMQ	☐	☐	☐	☑	☐	☐	Internal consistency: Cronbach's alpha; test–retest reliability: Spearman's correlation; differences between age and gender: ANOVA; EFA: Kaiser–Meyer–Olkin (KMO) and Bartlett's test; varimax rotation in SPSS.
Schulze et al. (2014)	PEDI‐G	☐	☐	☐	☑	☐	☐	R Core Team software was used for statistical analysis; ANOVA was used to compare ages. The ICC and Cohen's kappa coefficient, SEM and SDD were used for inter‐rater and test–retest analysis.
van der Linde et al. (2014)	DCDDaily‐Q	☐	☐	☑	☐	☐	☑	SPSS, nonparametric tests; internal consistency 0.7; discriminant validity, Mann–Whitney test used. Concurrent validity, calculated by Spearman's correlation; incremental validity, from binary logistic regression.
Mancini et al. (2016)	PEDI‐CAT/Brazil	☐	☐	☐	☑	☐	☑	For inter‐rater and test–retest reliability, ICC and CI were used; internal consistency (Cronbach's alpha). The structural equation modelling (SEM) was used for analysis of variance and the KMO index and Bartlett's test were used for EFA. The Kaiser criterion was used to define the number of factors, as well as oblique rotation.
Schulze et al. (2016)	PEDI‐G	☐	☐	☐	☑	☐	☑	Rasch method, using WINSTEPS software, including differential item functioning (DIF) and Mantel–Haenszel; differential test functioning (DTF); ANOVA for comparison between countries.
Ferreira et al. (2019)	DCDQ‐BR	☐	☐	☐	☐	☑	☑	Concurrent validity with the BOT‐2 instrument, using Pearson's correlation coefficient; predictive validity, sensitivity and specificity were calculated according to Portney and Watkins' proposal.
Mulligan (2019)	COPQ	☐	☐	☑	☐	☐	☑	Nonparametric comparison using the Mann–Whitney test for discriminant validity; Spearman's correlation was used for criterion validation.
Ray‐Kaeser et al. (2019)	DCDQ‐FE	☐	☐	☐	☑	☐	☑	Internal consistency (Cronbach's alpha), test–retest and inter‐rater reliability, ICC was used. The Mann–Whitney test, Chi‐squared test and Kruskal–Wallis test were used to compare groups. Spearman's correlation was used for concurrent validity.
Barrios‐Fernández et al. (2020)	ADL‐E	☐	☐	☐	☑	☐	☑	FACTOR software for data analysis; EFA for internal structure; KMO and Bartlett test; chi‐squared, CFI, NNFI and RMSEA were used for model adequacy. For internal consistency, ordinal alpha was used.
Delgado‐Lobete et al. (2020)	DCDDaily‐Q‐ES	☐	☐	☐	☑	☐	☑	Internal consistency, item homogeneity and construct validity. CFA, CFI, NNFI and RMSEA used.
Montes‐Montes et al. (2020)	DCDDaily‐Q‐ES	☐	☐	☑	☐	☐	☑	ANOVA to calculate the effect of age on participation, performance and score, t‐test to analyse gender differences; for concurrent validity: Pearson's correlation coefficient; ICC.
Montes‐Montes et al. (2020)	DCDQ‐ES	☐	☐	☐	☑	☐	☑	Construct validity: CFA, internal consistency; reliability calculated by Cronbach's alpha. Discriminant validity: Student's *t*‐test and ANOVA to analyse age and gender differences.
Moraes et al. (2022)	LDCDQ‐BR	☐	☐	☑	☑	☑	☑	To characterise and compare groups: SPSS, using nonparametric analyses; chi‐square and Mann–Whitney; JASP to estimate effect size; Rasch model to assess the quality of the items to classify the scale; reliability (Cronbach's alpha), test–retest, ICC, Spearman's correlation for concurrent validity; ROC curve, cutoff scores based on Youden index (J), sensitivity and specificity.

Abbreviations: ACHS, Assessment of Children's Hand Skills; ADL‐E, Activity Daily Living Spanish version; AMPS, assessment of motor and process skills; APCP, assessment of preschool children's participation; ASKp38, Activities Scale for Kids–performance; CFMQ, Contextual Fine Motor Questionnaire; CHSQ, Children's Hand‐Skills ability Questionnaire; COPQ, Children's Occupational Performance Questionnaire; DCDDaily‐Q, Developmental Coordination Disorder Questionnaire; DCDDaily‐Q‐ES, Developmental Coordination Disorder Questionnaire Spanish version; DCDQ‐BR, Developmental Coordination Disorder Questionnaire—Brazil version; DCDQ‐ES, Developmental Coordination Disorder Questionnaire Spanish version; DCDQ‐FE, Developmental Coordination Disorder Questionnaire French Europe version; LC‐MABC, Movement Assessment Battery for Children; LD‐CDQ‐BR, Little Developmental Coordination Disorder Questionnaire—Brazil version; LIFE‐H, Assessment of Life Habits; MAND, McCarron Assessment of Neuromuscular Development; PEDI‐CAT/Brazil, Paediatric Evaluation of Disability Inventory Computer Adaptive Test—Brazil version; PEDI‐G, Paediatric Evaluation of Disability Inventory—German version; VABS, Vineland Adaptive Behaviour Scales.

Among the methods used in the studies, reliability was the most prevalent (75%; *n* = 18). Cronbach's alpha and test–retest reliability measures using intraclass (ICC) were reported by 37.5% (*n* = 9). One study (0.24%) used ordinal alpha for internal consistency analysis (Barrios‐Fernández et al. 2020).

The content validation studies used panels of translators (when cross‐cultural adaptations were performed) and panels of experts, composed of up to nine professionals (van Der Linde et al. 2013). One study used the content validity index (CVI) and the Wilcoxon and Friedman tests in this content stage. Eleven studies (45.8%) reported Pearson/Spearman correlation for concurrent criterion validity.

Exploratory or confirmatory factor analysis, which is quite robust and recommended in validation studies, was reported by only 29.2% (*n* = 7) of the 17 studies that performed internal structure (construct) validation, highlighting the robust methodological approach of the Barrios‐Fernández et al. (2020) study, which employs rigorous psychometric standards. It was found that 12 studies (50%) used parametric (*t*‐test, analysis of variance—ANOVA) and nonparametric (Wilcoxon–Mann–Whitney, Kruskal–Wallis) analyses.

## 4 Discussion

Making mistakes in the first step (content validation) can generate negative consequences for both research and care (DeVellis 2012). Regarding the analysis of the content of the studies selected in this review (e.g., item wording and scale format), it is worth noting that it was not part of the objectives of this study to judge their quality. However, it is believed that a critical look is necessary when developing or adapting an instrument, and criteria such as objectivity, simplicity, clarity, precision, relevance and interpretability should be taken into account, with the aim of eliminating items that may be ambiguous, incomprehensible, with vague terms or double questions (Johnson and Morgan 2016; Coluci et al. 2015). To achieve this, it is essential to revisit the framework proposed by Johnson and Morgan (2016) and the Standards for Educational and Psychological Testing (AERA 2014). This process begins with best practices for writing and evidence based on test content, where the relationship between the test's content and the construct it is intended to measure is scrutinised.

Regarding the methods used to verify the evidence of content validity in the studies (assessment of consensus among experts), none of the studies cited the content validity ratio (CVR), which is considered one of the most widely used methods to establish this type of validity (Wilson et al. 2012). One of the studies reported using the CVI and the Wilcoxon and Friedman tests in this content stage; however, there are criticisms regarding the use of the CVI, whose results could be inflated and consequently could generate unreliable results (Polit and Beck 2006). Selecting the wrong technique may undermine the entire validation process, as the challenge at each stage is determining the appropriate moment to apply each method (Ferretti‐Rebustini 2023).

As for the other evidence of validity, face validity is considered the simplest, but least sophisticated and least satisfactory (Hair et al. 2005; Martins 2006); this type of validity was found in only one of the selected studies (Ramalho et al. 2013). The response process (pretest) was observed in the stage after the development of an instrument, as cited in the study by van Der Linde et al. (2013). This stage is considered important because it allows for the validation of evidence on the cognitive process, ensuring that the respondents' interpretation of items aligns with the intended construct (AERA 2014).

The internal structure (construct validity) and the relationship with other variables (criteria) are sometimes confused, and the methods used in the studies found in this review were sometimes incomplete and confusing regarding the type of validity. It is worth noting that the internal structure represents the extent to which the set of measured variables actually represents the latent variable they are designed to measure; that is, this type of validity seeks to present the true theoretical meaning of a concept (Martins 2006; Sampieri et al. 2006). According to Hair et al. (2009), this type of validity is determined by exploratory and confirmatory factor analysis, which was reported in seven studies (29.1%) described in this review. These factor analytical procedures, rooted in CTT, serve as a foundational step to verify dimensionality, which is also a prerequisite for the accurate application of IRT models. This type of analysis is performed using software such as JASP and FACTOR.

As for the relationship with other variables (criteria), it was observed that all studies that performed this type of validity related the instrument to other instruments. The use of ‘gold standard’ instruments is crucial for establishing convergent validity, as they provide a reliable baseline for comparison. No relationship was observed between the instrument and other variables, based on factors that could be associated with that particular condition, for example, socioeconomic level, presence of another comorbidity and other possibilities that could influence the result of the responses. However, this is a possibility to be carried out, especially if there is no reliable evidence from other instruments that measure the same measurement (AERA 2014). Half of the studies also cited parametric (*t*‐test, analysis of variance—ANOVA) and nonparametric (Wilcoxon–Mann–Whitney, Kruskal–Wallis) analyses when comparing groups, this type of analysis being cited by Johnson and Morgan (2016), one of the most contemporary references in psychometrics.

An in‐depth analysis of the results reveals the coexistence of different psychometric approaches: CTT and IRT. Although CTT focuses on the total test score and assumes that error is constant across the entire score range, IRT allows for a more granular evaluation by analysing the properties of individual items (difficulty and discrimination) (Hambleton et al. 1991). The choice between these models is based on their suitability for the analysis objectives and the characteristics of the sample, rather than a simple chronological succession. This review presents the two aspects of psychometrics, both CTT and IRT. Each type of validity evidence possesses a set of techniques that can be used and that have been improved over time; the challenge consists in knowing when to utilise each of them (Borsboom 2006, 2022; Ferretti‐Rebustini 2023).

It was observed that the studies that analysed reliability predominantly used coefficient tests related to CTT, such as internal consistency and test–retest. According to The Standards for Education and Psychological Testing (AERA 2014), there are limitations to these analyses, for example, when the same form of test is used (test–retest), the correlation between the two scores may be inflated due to the possible memory of the participant's initial responses. The authors also mention that coefficients based on IRT have advantages over this type of analysis and can, for example, be used to estimate standard error conditionally, depending on the level of the latent trait, rather than a single average value for the whole group as in CTT. This allows for a more sophisticated analysis of how well the instrument performs at different levels of severity or ability.

Given that the search was restricted to English and Portuguese (the authors' native language), a significant portion of the evidence for cultural adaptation and cross‐cultural validation stems from the Brazilian context. It is important to clarify that this does not imply ‘special treatment’, but rather a methodological necessity that will also impact future studies. Finally, regarding the nomenclature used in the types of validity evidence, only one study (Barrios‐Fernández et al. 2020) referred to terms used as currently recommended by The Standards for Education and Psychological Testing (AERA 2014), reinforcing the need for greater alignment with international psychometric guidelines.

## 5 Limitations

This study has some limitations. The manual search produced only two more articles, not including other measurement instruments such as the original version of the PEDI (nor the Brazilian version), although the German version, PEDI‐G, found through the electronic search, was included. In addition, when performing the search, instruments that did not present only the latent variable of child occupational performance were included, aiming to contemplate the largest number of instruments with mostly similar objectives, such as performance in ADLs and children's abilities in functional tasks.

## 6 Conclusions

This review allowed some conclusions: (1) The studies found provide an overview of validated performance assessment instruments related to the child population with DCD or other disabilities, with 17 different scales being found, four of which were adapted for the Brazilian version; (2) the content of the instruments presented variability in relation to the number of items (between 10 and 442), scale format (3 to 9 points) and domains evaluated, highlighting the latent variable most reported in the studies: ‘performance in ADLs’; (3) the types of validation performed varied between the studies, highlighting reliability, discriminant and construct validity; (4) the validation techniques used varied between the studies, and the methods used were sometimes incomplete and confusing; and little information was observed about the content validation process of the instruments; (5) although only reported by seven studies (29.2%), the presence of exploratory and confirmatory factor analysis methods was noted, considered a more robust technique in internal structure validation studies; (6) the use of the CVR was not observed in any of the content validation studies, which is considered the most recommended method for this type of validation; (7) an insignificant result was observed in relation to the use of nomenclatures in relation to the evidence of validity according to The Standards for Education and Psychological Testing (AERA 2014); (8) finally, the scarcity of originally Brazilian instruments and even cross‐culturally adapted instruments with the focus of evaluating the occupational performance of children, especially those with DCD, is notable.

## Author Contributions

**Jorge Alberto de Oliveira:** conceptualization, writing – review and editing. **Flávio Rebustini:** conceptualization, writing – review and editing, supervision, methodology. **Renata Hydee Hasue:** conceptualization, formal analysis, supervision, writing – review and editing, writing – original draft, methodology. **Carolina Barbosa de Souza:** formal analysis, investigation, data curation. **Marcela Favilla:** conceptualization, methodology, data curation, formal analysis, writing – original draft, resources, investigation, validation.

## Funding

This study was supported by the Coordenação de Aperfeiçoamento de Pessoal de Nível Superior, 88887.505615/2020‐00, 88887.816847/2023‐00.

## Conflict of interests

The authors declare no conflicts of interest.

## Data Availability

The data that support the findings of this study are openly available in Biblioteca Digital USP at https://www.teses.usp.br/teses/disponiveis/5/5170/tde‐14102025‐123832/, reference number https://www.teses.usp.br/teses/disponiveis/5/5170/tde‐141020.
